# Gait in patients with symptomatic osteoporotic vertebral compression fractures over 6 months of recovery

**DOI:** 10.1007/s40520-019-01203-9

**Published:** 2019-04-27

**Authors:** Eva Jacobs, Christopher McCrum, Rachel Senden, Lodewijk W. van Rhijn, K. Meijer, Paul C. Willems

**Affiliations:** 1 Department of Orthopaedic Surgery, Research School CAPHRI, Maastricht University Medical Center, P. Debyelaan 25, 6229 HX Maastricht, The Netherlands; 2 Department of Nutrition and Movement Sciences, NUTRIM School of Nutrition and Translational Research in Metabolism, Maastricht University Medical Center, Maastricht, The Netherlands; 3 Institute of Movement and Sport Gerontology, German Sport University, Cologne, Germany

**Keywords:** Spatiotemporal gait parameters, OVCF, Gait analysis, Vertebral fracture, Ageing spine

## Abstract

**Background:**

One factor related to disability in people with spinal deformity is decreased postural control and increased risk of falling. However, little is known about the effect of osteoporotic vertebral compression fractures (OVCFs) and their recovery on gait and stability. Walking characteristics of older adults with and without vertebral fractures have not yet been compared.

**Aims:**

The purpose of the current study was to examine the spatiotemporal gait parameters and their variability in patients with an OVCF and healthy participants during treadmill walking at baseline and after 6 months of recovery.

**Methods:**

Twelve female patients suffering a symptomatic OVCF were compared to 11 matched controls. Gait analysis was performed with a dual-belt instrumented treadmill with a 180° projection screen providing a virtual environment (computer-assisted rehabilitation environment). Results of patients with an OVCF and healthy participants were compared. Furthermore, spatiotemporal gait parameters were assessed over 6 months following the fracture.

**Results:**

Patients suffering from an OVCF appeared to walk with significantly shorter, faster and wider strides compared to their healthy counterparts. Although stride time and length improved over time, the majority of the parameters analysed remained unchanged after 6 months of conservative treatment.

**Discussion:**

Since patients do not fully recover to their previous level of mobility after 6 months of conservative treatment for OVCF, it appears of high clinical importance to add balance and gait training to the treatment algorithm of OVCFs.

**Conclusions:**

Patients suffering from an OVCF walk with shorter, faster and wider strides compared to their healthy counterparts adopt a less stable body configuration in the anterior direction, potentially increasing their risk of forward falls if perturbed. Although stride time and stride length improve over time even reaching healthy levels again, patients significantly deviate from normal gait patterns (e.g. in stability and step width) after 6 months of conservative treatment.

**Electronic supplementary material:**

The online version of this article (10.1007/s40520-019-01203-9) contains supplementary material, which is available to authorized users.

## Introduction

One of the most common fractures among older adults is osteoporotic vertebral compression fractures (OVCFs), with an estimated 500,000 new fractures occurring every year in Europe [1, 2]. After an OVCF, there is disproportionate height loss from the anterior vertebral body resulting in a wedge-shaped vertebra [3]. The presence of an OVCF is a strong predictor for subsequent vertebral fractures [4]. Wedge accumulation over multiple thoracolumbar levels may lead to thoracolumbar hyperkyphosis. Subsequent sagittal malalignment with inability to stand upright is the strongest driver of pain and disability in older adults suffering from spinal deformity [5, 6]. In the majority of the patients the initial pain caused by the fracture usually subsides within a couple of weeks, with healing of the fracture [2]. However, up to one-third of patients experience incapacitating pain for months to years due to insufficient response to treatment [2, 7].

One factor related to disability in people with spinal deformity such as thoracolumbar hyperkyphosis is decreased postural control and an increased risk of falling [8, 9]. Falls among older adults, especially those with osteoporosis, are associated with high morbidity, even mortality and involve high costs for society [8–10]. De Groot et al. [8] described that patients with an OVCF and thoracolumbar hyperkyphosis demonstrate impaired postural control. This may be due to the fact that in thoracolumbar hyperkyphosis, there is an increased forward bending moment due to the forward curvature of the trunk, which shifts the body’s centre of mass forward relative to the centre of rotation, i.e. the spine. In this situation, it may be more difficult to keep the centre of mass within the base of support (a requirement for stability in static situations) [11]. However, as the majority of falls in older adults occurs during walking, stability control specifically while walking should be considered. It is important to note that stability control during static tasks shows little relationship with more dynamic and reactive tasks [12–14]. In dynamic situations such as walking, the velocity of the centre of mass must be accounted for when assessing stability [11, 15]. For this purpose, Hof et al. [11] proposed the extrapolated centre of mass concept, in which the position and velocity of the centre of mass are taken into account when determining the stability of the body position. As people with thoracolumbar hyperkyphosis already have a more anterior centre of mass, stability in the anterior direction during walking may be reduced, perhaps increasing the difficulty in coping with forward losses of balance (e.g. trips) [8, 9]. Gait variability is another quantifiable feature of walking that is altered in clinically relevant syndromes or symptoms, such as falling, frailty and neuro-degenerative diseases. In literature, it has been demonstrated that increased gait variability is associated with an increased risk of falling [16]. However, there is little information available on the effect of OVCF on gait stability and variability.

The current study aimed to examine the spatiotemporal gait parameters and their variability in patients with an OVCF and healthy participants during treadmill walking. Furthermore, this study aimed to monitor recovery of spatiotemporal gait parameters and variability in patients suffering an OVCF over 6 months following the fracture. It was hypothesized that gait would be more variable in people with an osteoporotic vertebral fracture and that stability in the anterior direction would be reduced compared with healthy participants. Regarding the longitudinal data, it was hypothesized that gait would improve over time in terms of variability and stability.

## Materials and methods

### Participants

Twelve female patients [mean age 68 (55–78) years, mean height 1.61 m (1.51–1.73 m), weight 67.34 kg (49–88 kg)] with a symptomatic OVCF who presented at the emergency department of the Maastricht University Medical Centre gave a written informed consent to participate in this study. Participants were included if they were female, aged 55 years or older and were fully ambulatory (able to perform a 15-m walk test without assistance). Patients were excluded if the vertebral fracture was unstable and required surgery, if they had neurological deficits, active/recent cancer, a psychiatric diagnosis, a history of neurogenic or myopathic disorders impairing sensory or motor function or medication that could affect balance or walking. The study was explained before obtaining informed consent and conducted in accordance with the Declaration of Helsinki and was approved by the Maastricht University Medical Centre medical ethics committee (NL52978.057.15). Patients were treated with an orthosis (Osteolind^®^ plus, Werkmeister, Wanfried, Germany) which they wore all day for the first 6 weeks. From 6 weeks to 6 months, wearing the orthosis was optional. Data were collected at baseline, after 6 weeks and after 6 months.

A control group with gender, age, height and weight matched, was selected from the database of a previous study in the Maastricht University Medical Centre (NL58205.068.16) with 11 healthy older female adults [71 years (66–78 years), 1.64 m height (1.58–1.70 m) and 68.5 kg (62–81 kg) weight]. The participants had no self-reported history of walking difficulties, dizziness or balance problems, could walk for at least 30 min without assistance, without stopping and had no known neuromuscular condition, injury or medication that could affect balance or walking. Unpaired *t* tests confirmed that the groups were not significantly different in age, height or weight. Additionally, equivalence tests with 90% confidence intervals revealed that the group differences were statistically equivalent and not meaningfully different.

### Equipment

The measurements were conducted with the computer-assisted rehabilitation environment extended (CAREN; Motekforce Link, Amsterdam, The Netherlands), including a dual-belt instrumented treadmill (force plates: 1000 Hz), a 12-camera Vicon Nexus motion capture system (100 Hz; Vicon Motion Systems, Oxford, UK) and a virtual environment that provided optic flow. Participants wore a safety harness connected to an overhead frame. Ten retroreflective markers were attached to anatomical landmarks (one acromion marker on each side, four pelvic markers, one marker each lateral distal femur condyle and one marker on each lateral malleolus) and were tracked by the motion capture system. The marker trajectories were filtered using a low-pass second-order Butterworth filter (zero phase) with a cut-off frequency of 12 Hz. Foot touchdown and toe-off were determined using the treadmill force plates (50-N threshold) in combination with a marker-based method [17].

### Measurement procedure

Participants first completed treadmill walking familiarisation trials (consisting of 90-s walking at 1 m/s followed by 90-s walking at self-paced speed) after which they continued walking for 3 min at a self-paced mode followed by 3 min at a set speed of 1.0 m/s. For each of the 3 min, the first 90 s was unperturbed walking and the following 90 s included five mediolateral platform-shift perturbations. The perturbations were a part of another study and are not further discussed here. The final 60 strides of the 1-m/s walking trial were taken for further analysis, as well as the average walking speed during the unperturbed 90-s self-paced measurement as a functional outcome. In the OVCF group, the change in pain level was assessed using an 11-point visual analogue scale (VAS) where level 10 implies extreme pain and level 0 no pain at all.

### Healthy control data

Gait data from a walking trial at 1.0 m/s were taken for analysis. It is important to note that a slightly different reduced kinematic model was used for these subjects. For these participants, six markers were attached to each hallux, each trochanter major, the sacrum and the C7 vertebra. To determine the likely absolute differences of the two marker sets, we conducted a small pilot study with six participants wearing both marker sets. The protocol for these healthy pilot participants, and the details on the marker set comparability pilot study can be found in the supplement. The results section of the current study describes both the absolute data and the results accounting for the absolute differences found between the marker sets.

### Spatiotemporal gait and variability parameters

The gait parameters considered were stride time, stride length, step width and double support time. The mean value for each participant was calculated from all recorded steps. Additionally, the coefficient of variation (CV) was calculated for all gait parameters to evaluate gait variability. The margins of stability (MoS) were calculated in both the anteroposterior (AP) and mediolateral (ML) directions at foot touchdown as the AP or ML distance between the boundary of the base of support (BoS; the ankle marker) and the extrapolated centre of mass (*X*_CoM_), as defined by Hof et al. [11]. For the estimation of the centre of mass position and velocity, the average positions of the four pelvis markers were used. For the MoS the averages, medians and standard deviations were used for analysis.

### Statistics

Independent *t* tests were used to determine if differences existed between healthy control participants and patients with OVCF in all outcome parameters. To detect potential changes in the gait parameters in the patients over time, one-way repeated measures ANOVAs with Tukey post hoc tests for multiple comparisons were conducted. Statistical analyses were conducted with GraphPad Prism version 7.03 for Windows (GraphPad Software Inc., La Jolla, California, USA). Significance was set at *α* = 0.05.

## Results

### Baseline

The results for the spatiotemporal gait parameters at baseline (stride time, stride length, step width, double support time) of the OVCF group and the healthy controls are presented in Table 1. The independent *t* tests revealed a significantly shorter stride time, a significantly shorter stride length and a significantly greater step width for the OVCF group compared to the healthy participants, with no significant difference in double support time (Table 1). Additionally, the independent *t* tests revealed significantly lower AP MoS and significantly higher ML MoS for the OVCF group compared to controls (Table 1). All of the above differences exceeded any bias due to the difference in marker sets (Table 1; Supplementary Information). However, it is important to highlight that when accounting for the difference in the marker sets, the AP MoS is estimated to be on average 6 cm greater for the OVCF group (i.e. more stable anteriorly). No significant differences in the coefficients of variation for the four spatiotemporal gait parameters were found between the groups (Table 2).

**Table 1:** Spatiotemporal characteristics and margins of stability during walking at 1 m/s

	Stride time (s)	Stride length (m)	Step width (m)	Double support time (s)	MoS AP (m)	MoS ML (m)
OVCF	1.09 ± 0.05	1.10 ± 0.05	0.22 ± 0.03	0.13 ± 0.02	− 0.100 ± 0.034	0.048 ± 0.011
Control	1.17 ± 0.08	1.17 ± 0.08	0.14 ± 0.03	0.14 ± 0.02	0.105 ± 0.032	0.001 ± 0.009
*p* value	0.013*	0.024*	< 0.0001*	0.256	< 0.0001*	< 0.0001*
Exceeds bias of marker set difference?	**Yes**	**Yes**	**Yes**	N/A	**Yes**^a^	**Yes**

^a^This difference is higher than the upper confidence limit of the Bland–Altman plot, indicating that the OVCF group had a larger MoS AP value by on average 6 cm if the same marker set would have been used

* indicate *p* < 0.05

**Table 2:** Variability in the spatiotemporal characteristics and margins of stability during walking at 1 m/s

	Stride time (CV)	Stride length (CV)	Step width (CV)	Double support time (CV)	MoS AP (SD)	MoS ML (SD)
OVCF	2.09 ± 0.69	3.49 ± 2.47	11.87 ± 8.04	9.39 ± 3.22	0.019 ± 0.007	0.014 ± 0.004
Control	1.93 ± 0.62	2.90 ± 1.20	16.48 ± 5.48	10.49 ± 2.34	0.022 ± 0.007	0.013 ± 0.003
*p* value	0.556	0.480	0.126	0.361	0.331	0.754

### Longitudinal data

**Fig. 1:**
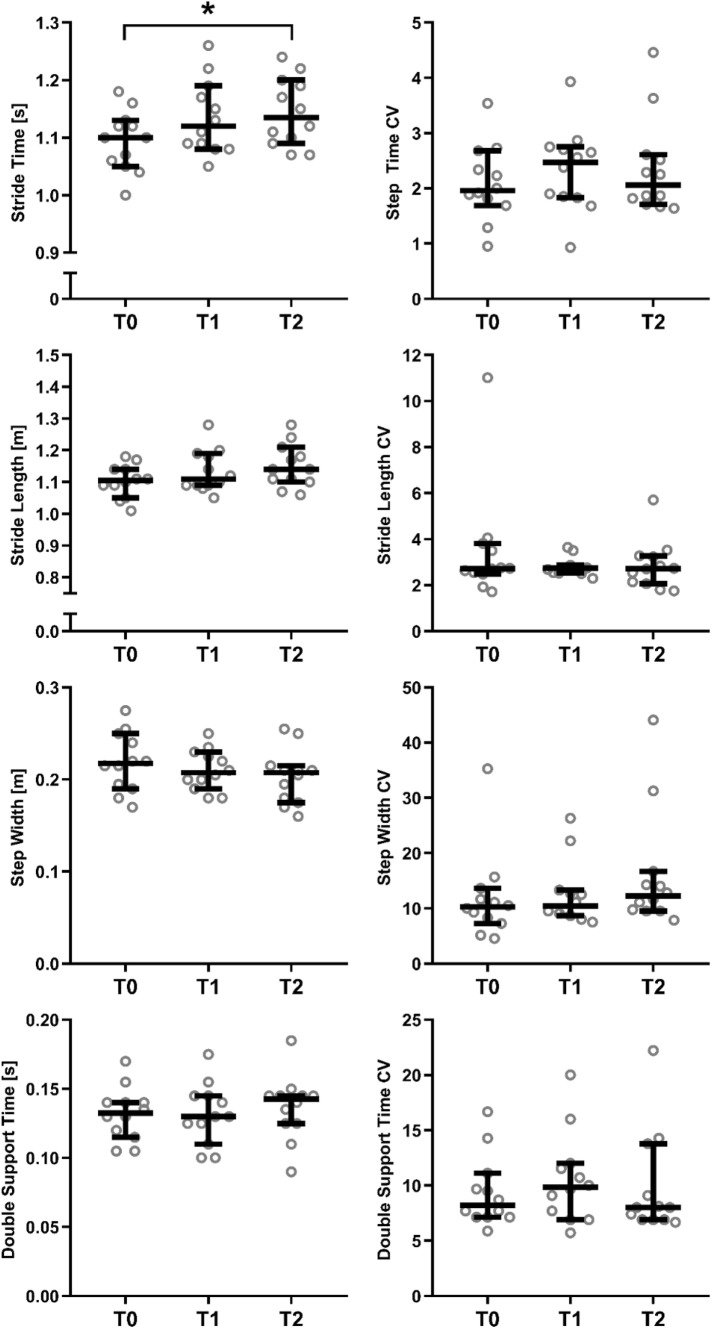
Stride time, stride length, step width and double support time in patients with an OVCF at baseline (T0), after 6 weeks (T1) and after 6 months (T2). Repeated measurements ANOVA demonstrated a significant improvement over time for stride time and length. There was no significant improvement in step width and double support time over time. Data presented for each patient, horizontal bar represents median with 95% confidence intervals

**Fig. 2:**
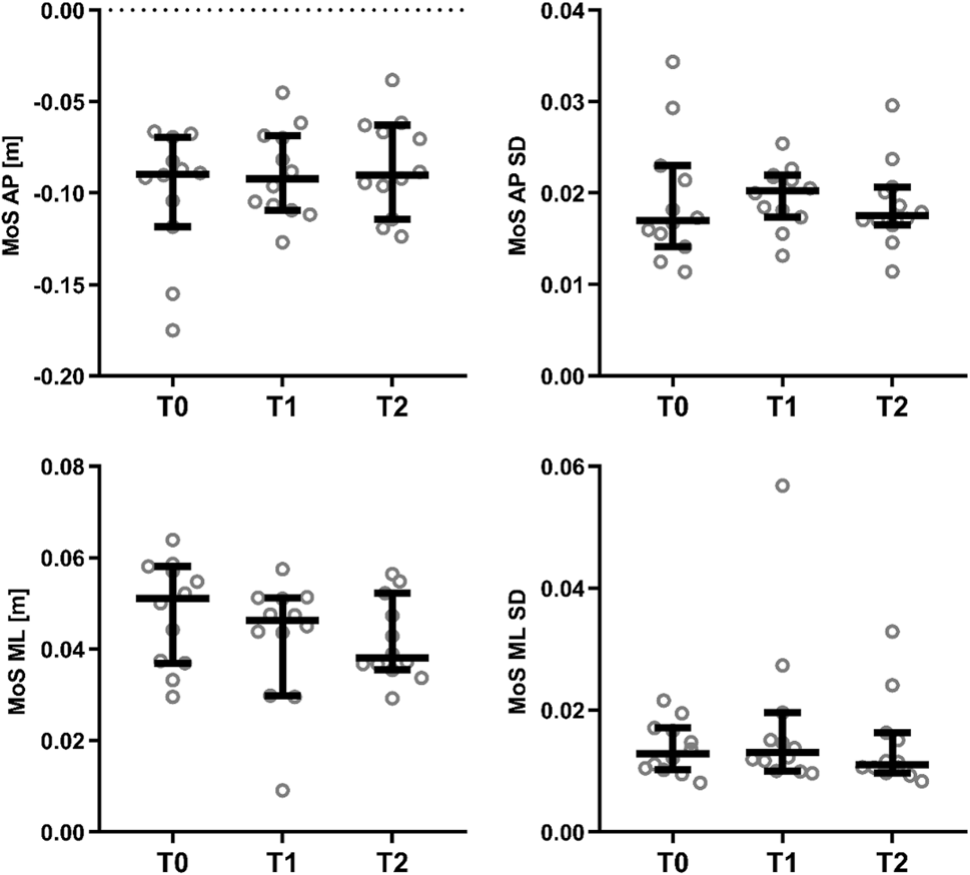
Margins of stability in the anteroposterior and mediolateral direction in patients with an OVCF at baseline (T0), after 6 weeks (T1) and after 6 months (T2). Repeated measures ANOVA demonstrated no significant differences over time. Data are presented for each patient; horizontal bar represents median with 95% confidence intervals

**Fig. 3:**
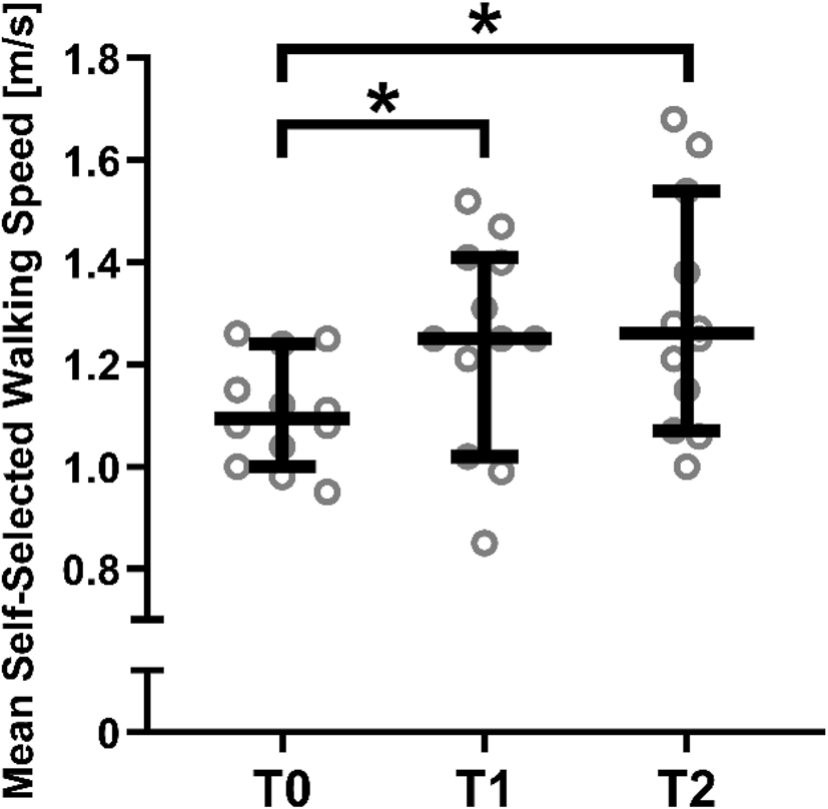
Self-selected walking speed in patients with an OVCF at baseline (T0), after 6 weeks (T1) and after 6 months (T2). There was a significant improvement over time. Data are presented for each patient; horizontal bar represents median with 95% confidence intervals

The repeated measures ANOVAs revealed significant effects of time on stride time (*F*_[1.2,14]_ = 6.4, *p* = 0.0191; Fig. 1) and stride length (*F*_[1.2,13]_ = 4.4, *p* = 0.0495; Fig. 1), and non-significant effects of time on step width (*F*_[1.9,21]_ = 2.4, *p* = 0.1178; Fig. 1), double support time (*F*_[1.6,17]_ = 1.7, *p* = 0.2182; Fig. 1), AP MoS (*F*_[1.3,14]_ = 2.5, *p* = 0.1269; Fig. 2) and ML MoS (*F*_[1.5,16]_ = 2.7, *p* = 0.1075; Fig. 2). For the significant effects, post hoc Tukey multiple comparisons tests revealed a significant difference between stride time (Fig. 1) at T0 vs. T2 (*p* = 0.0315) but no significant difference between T0 and T1 (*p* = 0.1045) or T1 and T2 (*p* = 0.4305). For stride length, no significant post hoc Tukey tests were found (*p* = 0.2779, *p* = 0.0751 and *p* = 0.0961 for T0 vs. T1, T0 vs. T2 and T1 vs. T2, respectively; Fig. 1). No significant effects of time were found for variability in stride time (*F*_[1.6,18]_ = 0.75, *p* = 0.4573), stride length (*F*_[1.2,13]_ = 0.79, *p* = 0.4136), step width (*F*_[1.5,17]_ = 3.3, *p* = 0.0742), double support time (*F*_[1.8,20]_ = 1.1, *p* = 0.3606) (Fig. 1), AP MoS (*F*_[1.8,20]_ = 0.14, *p* = 0.8522) or ML MoS (*F*_[1.3,15]_ = 0.8, *p* = 0.4206) (Fig. 2). As two parameters showed significant improvements over time, the T2 values of stride time and length were statistically compared to the healthy control data using independent *t* tests, which revealed no significant differences for stride time (*p* = 0.397) and stride length (*p* = 0.550). Regarding the mean self-selected walking speed from the 90-s period, there was a statistically significant effect of time on walking speed (*F*_[1.6,18]_ = 8.1, *p* = 0.0046; medians of 1.095 m/s, 1.250 m/s and 1.260 m/s for T0, T1 and T2, respectively; Fig. 3), with post hoc Tukey tests revealing significantly faster walking speeds at T2 (*p* = 0.018) and T1 (*p* = 0.041) compared to T0, but not between T1 and T2 (*p* = 0.409). Compared to baseline, patients showed significantly decreased VAS-pain scores 6 weeks and 6 months after fracture indicating less pain (VAS pain 5.17 + 1.64, 1.83 + 1.47 and 1.83 + 1.53, respectively; *p* < 0.05). Remarkably, four patients with disabling pain after 6 months of recovery showed no or little improvement in gait and stability over time, indicating that pain plays an important role in the recovery of gait after suffering an OVCF.

## Discussion

The current study primarily aimed to determine differences in spatiotemporal gait parameters and their variability between patients with an OVCF and healthy age-, gender-, height- and weight-matched participants during treadmill walking. The second aim was to assess whether those parameters and their variability changed over 6 months following the fracture. In contrast to our first hypothesis, the patients suffering an OVCF did not demonstrate more variable gait compared to the healthy controls. However, patients with an OVCF did walk with shorter, faster and wider strides at the given speed of 1 m/s compared to the healthy controls. These differences in stride length and time were no longer present 6 months after the fracture, indicating a return to a “healthy” stride time and length. Walking speed also improved after 6 months of conservative treatment. However, step width and both AP and ML margins of stability did not recover over time.

This is the first study in literature to elucidate the spatiotemporal gait characteristics and their variability of older adults suffering an OVCF. Hence comparison with previous studies is limited. In the context of this investigation, patients suffering an OVCF demonstrated shorter, faster and wider strides than healthy age-matched controls. These differences are also reported between young and older healthy adults, which may represent limitations in the ability to produce equivalent step lengths due to differences in muscle strength or physical capacity [18]. However, in the case of the current study, it might be speculated that the difference found in spatiotemporal gait parameters at baseline between the OVCF group and healthy controls might be due to pain. The differences in stride time and length at T0 and the lack of differences at T2 coincide with the healing of the fracture and with a significant decrease in pain of the patients. Given that those four patients who still had disabling pain at 6 months also showed a lack of improvement in the gait parameters suggests that an association exists between pain and (recovery of) gait after suffering an OVCF. Venmans et al. [19] found that 60% of conservatively treated patients with acute OVCFs had sufficient pain relief and good functional recovery within approximately 3 months after the acute fracture. However, 40% of patients still had disabling pain after 1 year [19, 20]. Especially, these patients should be screened for a history of falls or balance impairment and should follow appropriate (preventative) balance or gait training.

In a study by Hausdorff [16], stride time CV appeared to be a useful parameter to assess the risk of falling in elderly. In the current study, no significant difference in stride time CV was found between the OVCF group and healthy controls. This is in accordance with the study of de Groot et al. [8], in which only patients with a flexed posture demonstrated a greater variability in stride time in comparison with patients with a normal posture, whereas there were no differences between the groups in the presence of vertebral fractures. However, although there was no significant difference in stride time CV, in the current study vertebral fractures did seem to lead to alterations in AP and ML MoS, both with relatively greater stability than the control participants (when accounting for the marker set differences). The increased ML MoS corresponds with the significantly wider steps observed in the OVCF group. A wide base of support during gait is typically indicative of poor or cautious dynamic balance control [21]. However, the shorter stride length but slightly increased AP MoS (when accounting for marker set differences) implies that it was not the change in base of support that affected the MoS (as the value would then be smaller in the patients), but rather that the centre of mass position and/or accelerations that were less anterior in the OVCF group. This may represent a compensation for pain or discomfort to reduce fluctuations in the upper body. The likelihood that the AP MoS difference is mainly due to the centre of mass characteristics is supported by the longitudinal data, as despite an increased stride length over time (and, therefore, a larger base of support) these changes are not reflected in the AP MoS.

Gait is an important indicator of health and can be influenced by many variables [16, 22–24]. Older adults with chronic low back pain exhibit significantly different gait patterns compared to age- and sex-matched adults without chronic low back pain [25]. In the current study, it was demonstrated that only two of all measured gait parameters improved and returned to “healthy” levels after 6 months of conservative treatment (self-selected gait speed and stride time at 1 m/s). Most parameters, including step width, double support time and MoS, remained unchanged (Fig. 1). These parameters are closely related to gait stability. Therefore, it appears that overall, the capacity of the patients’ gait in terms of stride length and speed improved over time, but deficits in stability did not. This may imply that the OVCF population is at a greater risk for incident disability, such as falling, than their healthy counterparts even 6 months after conservative treatment [24]. These findings suggest that an OVCF can be the serious beginning of a downward cascade which should be taken into account in the multimodal treatment of vertebral fractures and osteoporosis.

An important limitation of this study is the small number of subjects that were included, which may have affected the power of the study. However, as patients suffering an OVCF are often unable to participate due to incapacitating pain and inability to walk independently, together with the fact that these patients are typically characterised by a combination of physiological, psychological and social problems, gait and stability studies on this specific condition will always be faced with this challenge. However, the individual data points and the changes over time do appear to confirm the statistical outcomes and so we believe that this may not have affected the overall conclusions of the study. A second limitation is the lack of consistent marker sets in the patient and control groups. However, the pilot study with 360 data points in the Bland–Altman plots addressed this limitation providing sufficient data to estimate the absolute difference in the marker sets. Furthermore, the longitudinal analysis has not been affected by this limitation as the patients were measured every time with the same marker set.

In conclusion, the results of the current study indicated that patients suffering from an OVCF walk with shorter, faster and wider strides compared to their healthy counterparts adopt a less stable body configuration in the anterior direction, potentially increasing their risk of forward falls if perturbed. Although stride time and stride length improve over time even reaching healthy levels again, patients significantly deviate from normal gait patterns (e.g. in stability and step width) after 6 months of conservative treatment.

## Electronic supplementary material

Below is the link to the electronic supplementary material.

Supplementary material 1 (DOCX 313 kb)
