# Supplementary material for: Gait in patients with symptomatic osteoporotic vertebral compression fractures over 6 months of recovery
Source: Aging Clin Exp Res. 2019 Apr 27;32(2):239–46. doi: 10.1007/s40520-019-01203-9 (PMC7033073; doi:10.1007/s40520-019-01203-9)
Supplement: Supplementary file 1 — Supplementary material 1 (DOCX 313 kb) [file 40520_2019_1203_MOESM1_ESM.docx]

**Supplementary Information**

Marker Set Comparison Pilot

*Rationale*

In order to determine the differences between groups in the main study that could simply be due to absolute differences in the two different reduced marker sets, this pilot study conducted measurements of participants walking while wearing both marker sets. In this way, we could estimate the absolute difference in the parameters of interest due to the marker sets and determine if the differences found in the main study exceeded these.

*Methods*

6 healthy young adults (age [mean±SD]: 23.3±3.7y; height: 175.6±6.4cm; weight: 66.5±9.1kg) participated in the pilot measurements. These measurements were registered with the medical ethics committee of Maastricht University (number: 2018-0568; METC azM/UM), written informed consent was obtained from participants and the measurements were conducted in accordance with the Declaration of Helsinki.

16 retroreflective markers were attached to anatomical landmarks of the participants comprising both marker sets used in the main study (see main text for details). Participants walked for three minutes at 1m/s on the Computer Assisted Rehabilitation Environment Extended (CAREN; Motekforce Link, Amsterdam, The Netherlands), as in the main study. In post-processing, copies of the motion capture data were made and marker coordinates were removed for each of the marker sets respectively so that one file contained only the 6 marker set and the other contained only the 10 marker set for the same motion capture recording. Following this, the final 60 strides from each recording were analysed for the gait parameters stride time, stride length and double support time, with the final 60 steps analysed for step width. Additionally, the margins of stability (MoS) were calculated in both the anteroposterior (AP) and mediolateral (ML) directions at foot touchdown for the final 60 steps as the AP or ML distance between the boundary of the base of support (BoS; the hallux marker) and the extrapolated centre of mass (XCoM), as defined by Hof *et al*. (2005), adapted for the reduced kinematic model based on Süptitz *et al*. (2013), whereby the position of the centre of mass was the average of the trochantor makers and the velocity of the centre of mass was estimated as:

$0.5\left( \frac{V_{\mathrm{TroL}}+V_{\mathrm{TroR}}}{2}+V_{C7} \right)$

where $V_{\mathrm{TroL}}$ , $V_{\mathrm{TroR}}$ and $V_{C7}$ are the anteroposterior or mediolateral velocities of the trochanter and C7 markers respectively. For the AP direction, the velocity of the treadmill belt was added.

*Results*


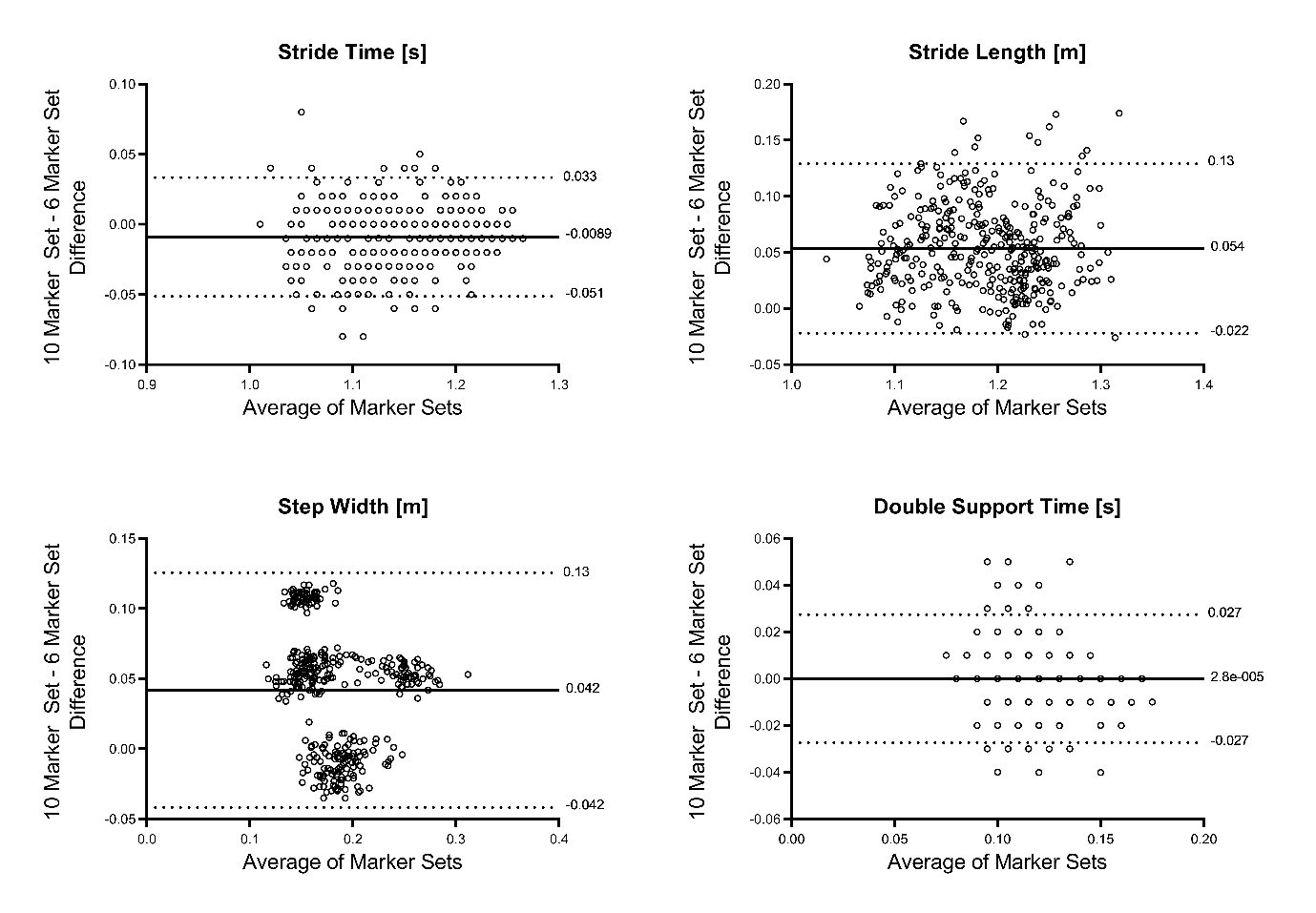


Supplementary Figure 1: Bland-Altman plots of the spatiotemporal parameters stride time, stride length, step width and double support time from 60 strides (or steps for step width) from six young healthy participants (total trials n=360). Mean bias is indicated by the solid black line and upper and lower 95% limits of agreement are indicated by the dotted lines.


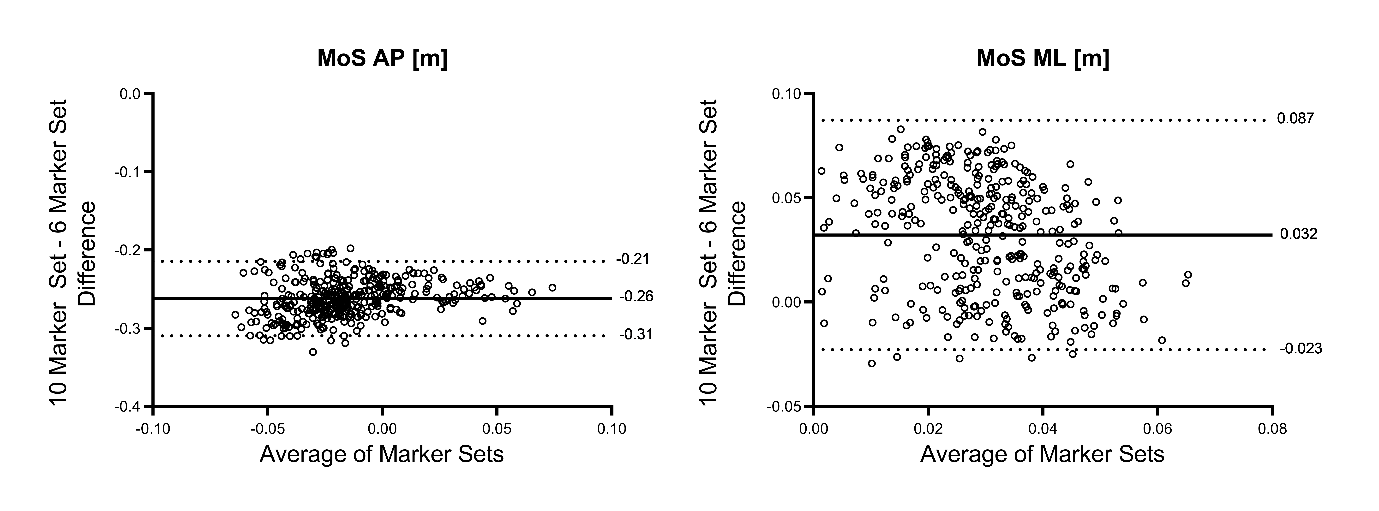


Supplementary Figure 2: Bland-Altman plots of the margins of stability in the anteroposterior direction (MoS AP) and the mediolateral direction (MoS ML) from 60 steps of six young healthy participants (total trials n=360). Mean bias is indicated by the solid black line and upper and lower 95% limits of agreement are indicated by the dotted lines.
